# Quinolone Complexes with Lanthanide Ions: An Insight into their Analytical Applications and Biological Activity

**DOI:** 10.3390/molecules25061347

**Published:** 2020-03-16

**Authors:** Ana-Mădălina Măciucă, Alexandra-Cristina Munteanu, Valentina Uivarosi

**Affiliations:** Department of General and Inorganic Chemistry, Faculty of Pharmacy, Carol Davila University of Medicine and Pharmacy, 6 Traian Vuia St, Bucharest 020956, Romania; mada.maciuca@yahoo.com (A.-M.M.); alexandra.ticea@umfcd.ro (A.-C.M.)

**Keywords:** fluoroquinolones, lanthanides, metal complexes, biological activity, quantitative determination

## Abstract

Quinolones comprise a series of synthetic bactericidal agents with a broad spectrum of activity and good bioavailability. An important feature of these molecules is their capacity to bind metal ions in complexes with relevant biological and analytical applications. Interestingly, lanthanide ions possess extremely attractive properties that result from the behavior of the internal 4f electrons, behavior which is not lost upon ionization, nor after coordination. Subsequently, a more detailed discussion about metal complexes of quinolones with lanthanide ions in terms of chemical and biological properties is made. These complexes present a series of characteristics, such as narrow and highly structured emission bands; large gaps between absorption and emission wavelengths (Stokes shifts); and long excited-state lifetimes, which render them suitable for highly sensitive and selective analytical methods of quantitation. Moreover, quinolones have been widely prescribed in both human and animal treatments, which has led to an increase in their impact on the environment, and therefore to a growing interest in the development of new methods for their quantitative determination. Therefore, analytical applications for the quantitative determination of quinolones, lanthanide and miscellaneous ions and nucleic acids, along with other applications, are reviewed here.

## 1. Introduction

Quinolones comprise a series of synthetic bactericidal agents with a broad spectrum of activity and good bioavailability [1,2], characteristics that make them suitable candidates for treating infectious diseases with various localizations: cutaneous, urinary, respiratory, bone, gastrointestinal, etc. [3].

**Figure 1:**
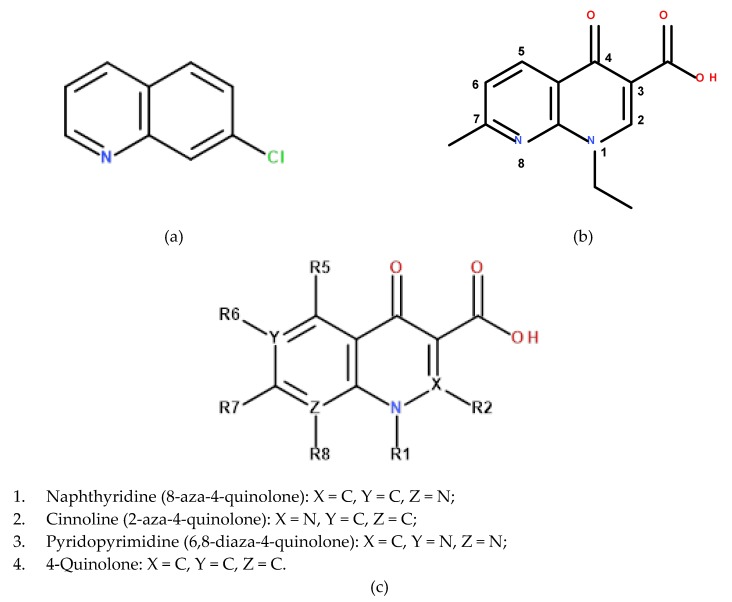
(**a**) 7-chloroquinoline; (**b**) nalidixic acid; (**c**) general structure and main classes of quinolones.

The starting point in the development of quinolones was the synthesis of nalidixic acid by G.Y. Lesher and coworkers in 1962, starting from 7-chloroquinoline (Figure 1a), a compound with antibacterial properties, a secondary product from the synthesis of chloroquine [4]. Nalidixic acid (Figure 1b) was proven to be active against certain Gram-negative pathogens, which led to the start of its clinical use in the treatment of urinary tract infections [5].

To date, numerous modifications have been brought to the nalidixic acid scaffold, which have resulted in a broader antibacterial spectrum, a different mode of binding to the plasmatic proteins and a longer half-time; significant changes have been obtained by the attachment of the fluorine atom in position 6 (fluoroquinolones) and a piperazine ring in position 7. Based on their chemical structures, these derivatives can be divided into four classes (Figure 1c): naphtyridine (nalidixic acid, enoxacin, gemifloxacin, tosufloxacin), cinnoline (cinoxacin), pyridopyrimidine (pipemidic acid, piromidic acid) and 4-quinolone (oxolinic acid, flumequine, norfloxacin, ciprofloxacin, ofloxacin, levofloxacin, sparfloxacin, etc.) [6]. Based on the antimicrobial spectrum and the pharmacological properties, four generations of quinolones are known. The improved properties of modern quinolones (4th generation) have rendered them suitable for treating more serious infections caused by particular pathogens (Gram-positive, resistant strains, anaerobe, etc.) with various localizations (respiratory, intra-abdominal, etc.) [7,8]. 

Quinolones possess a bactericidal mechanism of action which comes as a result of the inhibition of DNA transcription and replication in bacteria. In essence, in mycobacteria, and the majority of Gram-negative bacteria, quinolones act predominantly by disrupting the activity of topoisomerase II, also known as DNA-gyrase [9,10]; meanwhile in Gram-positive bacteria, topoisomerase IV is their primary target [11,12]. Although structurally related, the two enzymes play different roles in bacterial replication, a process known as binary fission. DNA-gyrase catalyzes the formation of negative supercoils in the circular, double-stranded bacterial DNA structure [13,14]. Topoisomerase IV is involved in the despiralization and decatenation of the double-stranded DNA molecule, preparing it for the transcription and replication processes, and separation of the DNA-daughter helices, facilitating their segregation into two daughter cells at the end of the replication process [15,16].

**Figure 2:**
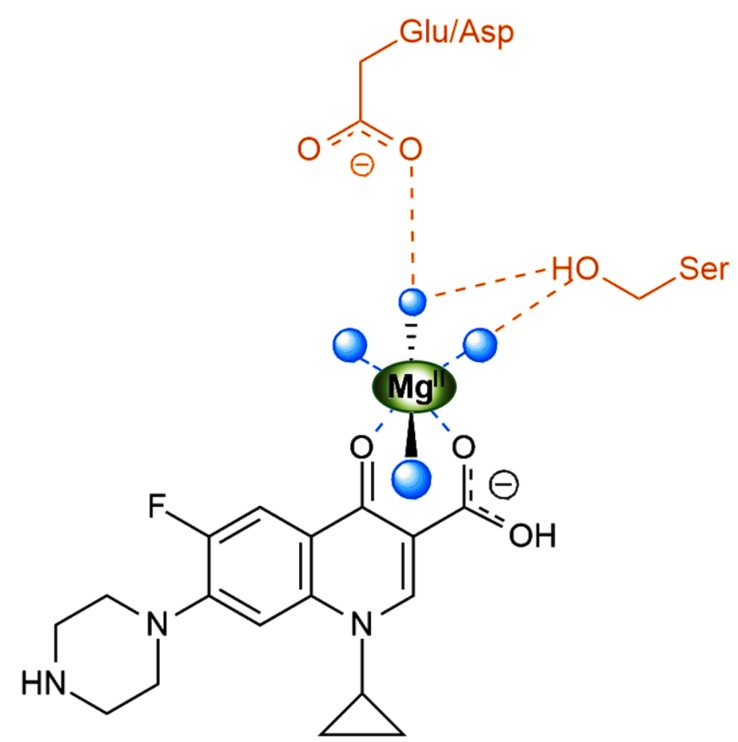
Coordination of Mg^2+^ at the binding site of ciprofloxacin to topoisomerase IV in the quinolone–DNA–enzyme complex; the four water molecules are represented through four blue bullets (adapted from [19], with permission https://pubs.acs.org/doi/10.1021/bi5000564; further permissions related to the material excerpted should be directed to the ACS).

Quinolones inhibit the activity of these two enzymes, not by direct binding, but through the formation of a quinolone–bacterial DNA–enzyme complex; this process disrupts DNA replication and consequently causes rapid cell death [17]. Magnesium ions are essential for the formation of the quinolone–DNA–enzyme complex [18]. In the proximity of the DNA binding site of the enzyme, Mg^2+^ is coordinated by two oxygen atoms from the quinolone molecules and four water molecules, in an octahedral arrangement (Figure 2) [19]. This process emphasizes the crucial importance of the 4-oxo (carbonyl) group for the antibacterial activity of quinolones.

Quinolone molecules, which possess a basic heterocyclic ring in position 7 (e.g., piperazinyl), display zwitterionic character. Between the pH values of 3 and 11, quinolones exist in the zwitterionic form, which is the most likely species to efficiently penetrate cell membranes; the completely protonated form (QH_2_^+^) exists in a proportion of 99.9% at the pH value of 1, while at pH = 7.4 all species are present in quantifiable proportions [20]. 

**Figure 3:**
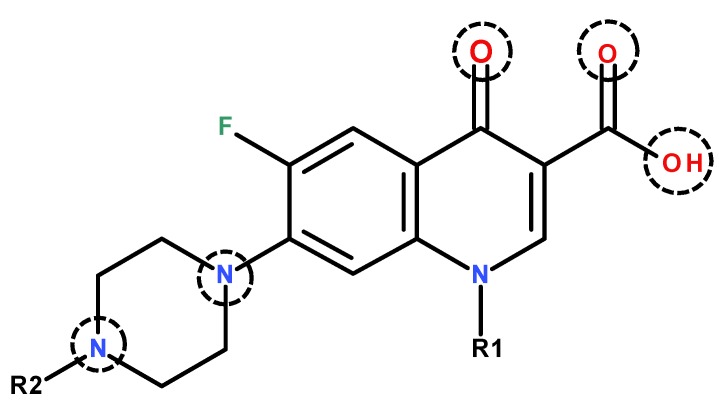
Donor atoms in the general structure of fluoroquinolones.

Worthy of note is that these molecules possess a remarkable capacity to bind metal ions, correlated with the donor atoms in their structures (Figure 3). Depending on the working conditions (pH, nature of the metal ion, etc.), quinolones can act as bidentate ligands (coordinated to the metal ion via one oxygen atom from the carboxyl group and the oxygen atom from the 4-oxo moiety; via both oxygen atoms of the carboxyl moiety; or through the nitrogen atoms of the piperazine ring). Rarely, a unidentate coordination has been reported [21] (Figure 3).

The ability of quinolones to form metal complexes has been explored in various ways. Numerous studies revealed a superior antimicrobial activity of the metal complexes compared to the free ligands, or even new biological activities (antifungal, antiparasitic or anticancer) [22,23,24,25,26,27]. Moreover, the quinolone–metal complexes are valuable in the battle against bacterial resistance, since they present higher lipophilicity due to the chelation effect [28] and offer a different mechanism of action, including their capacity to bind DNA. Metal complexation has found valuable analytical applications in the quantitative determination of both quinolones and metal ions.

## 2. Metal Complexes of Quinolones with Lanthanide Ions

A relatively new direction in the field of complex combinations of quinolones is represented by chelates with lanthanide trivalent metal ions (Ln^3+^). Lanthanides have become of interest to researchers after the observation that Ca^2+^ and La^3+^ are similar in terms of ionic radii, which led to the idea that La^3+^ can replace the former on different physiological binding sites, disrupting different physiological mechanisms [29]. Additionally, with the development of stable MRI contrast agents based on gadolinium (III) chelates [30,31,32] and the ability of Tb^3+^ and Eu^3+^ luminescent complexes to bind to the “drug site II” of serum albumin [33], the exploitation of chelating properties and luminescent complexes has begun.

The most important properties of lanthanides result from the behavior of the internal 4f electrons, behavior which is not lost upon ionization, nor after coordination. The strong electropositive character of lanthanides decreases with the drop in radius size, europium being the most reactive; likewise, the strong ionic trait of the compounds and the basic character of the hydroxides drop along the series, Yb(OH)_3_ and Lu(OH)_3_ being the least basic of all. Consequently, their aqua ions are the most predisposed to hydrolysis, phenomena that can be prevented by working in acidic conditions [34,35]. They predominantly form Ln^3+^ ions, but +4 and +2 oxidation states are also present in some cases (Ce^4+^, Pr^4+^, Eu^2+^).

**Figure 4:**
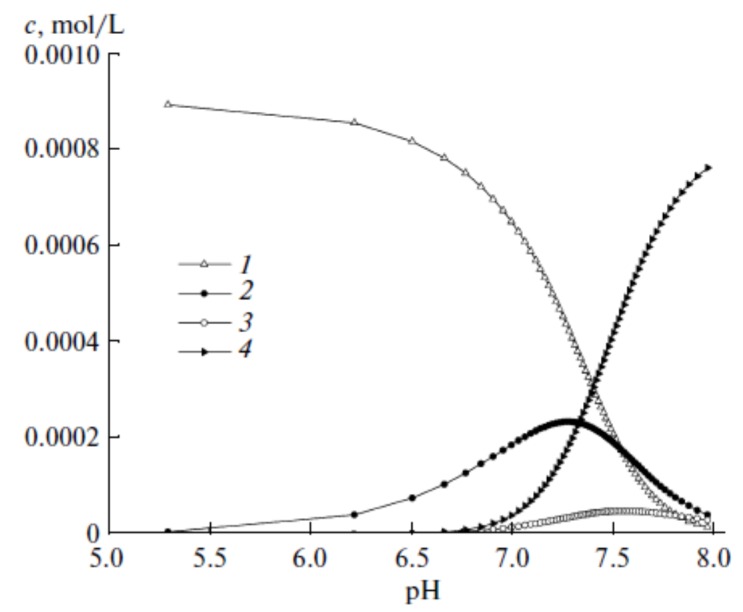
Equilibrium speciation distribution vs. pH for the Lu(NO_3_)_3_ system on the background of 0.1M KNO_3_: (1) Lu^3+^, (2) Lu(OH)^2+^, (3) Lu(OH)_2_^+^, (4) Lu(OH)_3_—with permission from [36].

The correlation between hydrolysis constants and the drop in ion radius among the lanthanide series has been experimentally proven. At the end of the titration, with the rising in pH value, opalescence was observed due to the formation of Ln(OH)_3_ colloid solutions. A distribution of the species correlated to the pH values is represented in Figure 4 [36]. These data should be taken into account when choosing the pH value for the medium used for a complexation reaction.

On the matter of complex formation, lanthanides are classified as “class A” or “hard” acceptors, and therefore prefer “class A” or “hard” donor ligands (in the order O > N > S and F > Cl). In aqueous solutions, lanthanide ions attract water molecules, forming hydration shells; the number of water molecules varies: higher coordination numbers are characteristic for lighter lanthanides, ([Ln(H_2_O)_9_]^3+^, Ln = La-Eu) and lower numbers for the heavier lanthanides ([Ln(H_2_O)_8_]^3+^, Ln = Dy-Lu). Eu^3+^ and Gd^3+^ exist as mixed populations, displaying both hydration numbers. This decrease in hydration number can be explained by the rise in energy in the bond between the ion and each coordinated water molecule; the bond is so strong that only a few ligands can compete and form complexes in aqueous solutions, chelates being more stable. Coordination numbers vary from 6 to 12 in general; biologically relevant coordination numbers are 8 or 9. Consequently, the complexes can have a variety of geometrical conformations. A coordination number of 10 requires ligands with “smaller bites,” such as NO_3_^−^ or SO_4_^2−^. Complexes with ligands with donor atoms other than oxygen are known, but they must be prepared in the absence of water [35].

Electronic transitions in the 4f orbitals of lanthanide ions give rise to characteristic absorption spectra. Only Y^3+^, La^3+^ and Lu^3+^ do not absorb in the UV or visible domain; all the other lanthanides have characteristic bands in this region, but the absorptivities have low values. The lanthanide ions with stronger fluorescence are Sm^3^^+^, Eu^3^^+^, Tb^3^^+^ and Dy^3^^+^, because the excitation energy level of these ions lies slightly lower than the excited levels of the ligands; thus they are able to readily accept the energy transfer [37]. A luminescent behavior suitable for detection and quantification can only be observed upon excitation by UV irradiation, X-rays, fast electrons, neutrons and certain chemical or mechanical means. It has been noted that certain molecules can enhance the luminescence of lanthanides upon complexation by indirect excitation via a suitable chromophore moiety in the complex [38].

When developing a synthesis method for quinolone–lanthanide complexes, one must take into account the zwitterionic nature of some quinolones and the pH value that is the most advantageous to the complexation process, and also correlate it to the pH value at which the lanthanide ion is stable, taking into account the hydrolysis tendencies it might have at a higher pH. Table 1 summarizes various synthesis methods used for obtaining solid lanthanide–quinolone complexes.

Analyzing the data presented in Table 1, some general remarks can be drawn as follows: the molar ratios used are 2:1 or in some cases 3:1 ligand to metal; several heteroleptic complexes have been reported, bearing as a second ligand 1,10-phenanthroline [48], DL-alanine [57] or glycine [49]. The pH value varies from 5 [40] to 9 [46]. The general scheme for synthesis is based on the following steps: the lanthanide salt and the quinolone are dissolved separately in different solvents (water, methanol, ethanol, acetone), and then the salt solution is added dropwise into the quinolone solution. The mixture is either kept under continuous stirring at room temperature for several hours (from 15 h [47] to 7 days [41]) or refluxed for different periods of time (from 2 h [45] to 8 h [43]). The obtained precipitate is removed from the reaction medium via filtration or centrifugation, washed and dried.

In the majority of these complexes, quinolone drugs behave as anionic, bidentate ligands coordinated to the metal ions via pyridone oxygen and carboxylate oxygen. In some complexes, quinolones are present in their zwitterionic form [45] or in both their anionic and zwitterionic forms [42]. The coordination mode via the carbonyl oxygen and hydroxyl oxygen of the carboxyl group [39] was also reported, while in a dinuclear complex with a nine-coordinated lanthanide ion, two anionic quinolone molecules acted as tridentate chelate and bridging ligands and two as bidentate chelate ligands [44].

## 3. Biological Activity of Metal Complexes of Quinolones with Lanthanide Ions

The biological activities of several metal complexes obtained in solid state have been assessed. Table 2 and Table 3 comprise data regarding the biological activity evaluations and calf-thymus DNA (CT-DNA) and bovine serum albumin (BSA) binding studies published for quinolone complexes with lanthanide ions, respectively. 

It was observed that the complexes have improved water solubility in comparison with the quinolone parent molecule. Thereby, the complexation may improve both the hydrophilic and lipophilic properties of quinolone, and give better bioavailability and antibacterial activity [41]. Liposolubility is an important factor for the antimicrobial activity. Upon complexation, the polarity of the metal ion is reduced due to the overlap with ligand orbitals and partial sharing of the positive charge of the metal ion with the donor groups. The increased liposolubility of the ligand upon metal chelation may contribute to its facile transport into the bacterial cell; once it has entered the cell as a metal complex, the ligand blocks the metal binding sites of crucial bacterial enzymes [50].

When taking into account the chemical structures of the metal complexes, the following five principal factors greatly influence their antimicrobial activities: (1) the chelate effect-bidentate ligands show higher antimicrobial efficiency towards complexes with monodentate ligands; (2) the nature of the ligand/ligands; (3) the total charge of the complex; the antimicrobial activity varies in the following order—cationic > neutral > anionic complex; (4) the nature of the counter ion for ionic complexes; (5) the nuclearity of a metal center in complex with a dinuclear center is more active than that of a mononuclear one [21].

Briefly, lanthanide complexes of quinolones have been tested in regard to their antibacterial, antifungal, antitumoral and/or antioxidant properties; in addition, in some cases, their ability to bind to calf-thymus DNA (CT-DNA) and bovine serum albumin (BSA) has also been quantified. The antibacterial activity was tested against *Escherichia coli, Staphylococcus aureus, Pseudomonas aeruginosa, Streptococcus pneumoniae* and *Bacillus subtilis;* meanwhile, the antifungal activity was tested against *Candida albicans* and *Aspergillus awamori.* As a general remark, the complexes showed no antifungal activity, except for the complex of Ce^4+^ with gemifloxacin [48]. The antibacterial activity of the complexes varies greatly, from being lower than the ligand [43], to being the same for some strains [39,47,48,51,57] and to being higher than the ligand [39,41,44,48,50,51]. Regarding the CT-DNA and BSA binding experiments, the complexes proved to have higher affinity than the ligand both for the former [52,55,58] and the latter [44,56]. 

## 4. Analytical Applications of Metal Complexes of Quinolones with Lanthanide Ions

### 4.1. Quantitative Determination of Quinolones

Due to quinolones being widely prescribed in both human and animal treatments, their concentration in nature has reached a critical level [59]. This led to an increasing interest in the development of new methods for their quantitative determination. The challenge is that these methods need not only address the determination of quinolones in pure state, pharmaceutical formulations and biological fluids, but also in water, soil and animal products, such as milk and meat. 

**Figure 5:**
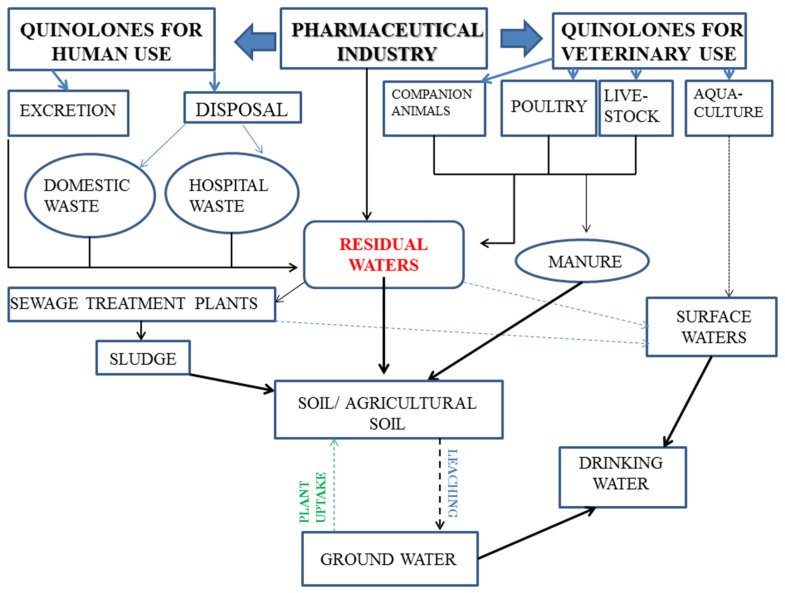
Main routes of contaminating the environment with quinolones for human or veterinary use (adapted after [59,63]).

Fluoroquinolones and their metabolites enter the environment after human or veterinary use, as a result of their excretion through urine or stool; after the human excreta enters sewage-treatment plants, most compounds reach the sewage sludges; some by-products are used to fertilize soils and others remain in water effluents. Alternate ways for fluoroquinolones to enter the water circuit are through treatment of the aquaculture and/or direct discharge. Although studies show that fluoroquinolones suffer photolysis [60] in the surface layers of water under direct exposure to UV radiation, adsorption or biodegradation, the phenomenon raises concerns about the environmental impact and the promotion of resistant strains [61]. Fluoroquinolones eliminated by animals end up in manure which is later used as fertilizer in agricultural soils (Figure 5). A discussion about how this process affects the soil has been made elsewhere [62]. Fluoroquinolones from domestic or hospital waste or from the manufacturing process are not considered relevant, as they are subjected to regulatory control. In some cases, however, the concentrations of antibiotics in hospital effluents are of the same order of magnitude as the minimum inhibitory concentrations [63].

This has challenged researchers to develop new methods for the quantitative determination of quinolones that are sensitive to concentrations of nanograms/mL [64] or micrograms/kg [65], and to adapt them in order to use complex matrices (e.g., food or soil) as probes.

Numerous methods have been reported for the determination of quinolones; namely, HPLC [66,67], capillary electrophoresis [68,69], MS spectrometry [70], voltammetry [71,72], polarography [73], titrimetry [74] and spectrophotometry [75,76]. Among them, spectrofluorometry and luminescence methods have been reported to be the most attractive to scientists, as they are highly sensitive and simple methods that require inexpensive equipment, and are suitable for the analysis of compounds in either pure state, pharmaceutical formulations or biological fluids. Various methods have been developed; e.g., native drug fluorescence measurements or fluorescence measurements after the formation of charge transfer complexes, chemometric methods, lanthanide sensitized fluorescence and chemiluminescent methods. Methods for the measurement of native fluorescence of quinolones in different systems or enhanced fluorescence due to complex formation with different metals have been reviewed by Singh et al. [77].

Due to their large conjugation systems and rigid structures, most quinolones absorb in the UV region and possess intrinsic fluorescence. Their UV spectra generally display two main peaks: one around 250 nm (strong) and one around 300 nm (weak). The excitation wavelength is set at around 300 nm in order to avoid the direct excitation of the lanthanide cation. It can be observed that upon addition of the lanthanide cation, the fluorescence intensity of the fluoroquinolone decreases as the specific bands of the Ln^3+^ cation appear. The energy absorbed by the quinolone molecule at its characteristic excitation wavelength is transferred to a triple state of the molecule and then intramolecularly transferred to a resonance level of the lanthanide ion, which finally emits luminescence at its particular emission wavelength. The efficiency of the transfer depends on the match of the ligand and lanthanide energy levels [78]. 

Lanthanide cations possess intrinsic photoluminescent properties. However, their poor ability to absorb light energy and their fast non-radiative deactivation by solvent molecules prevent the generation of luminescence by direct excitation. This issue can be resolved via luminescence sensitization or antenna effect, meaning chelation with appropriate organic ligands that enhance the spectroscopic properties of the lanthanide ions [79]. These chelates present a series of spectroscopic properties which render them suitable for analysis: narrow and highly structured emission bands; large gaps between absorption and emission wavelengths (Stokes shifts); and long excited-state lifetimes [80,81]. Once the chelate is formed, an intramolecular energy transfer takes place from the excited state of the ligand to the emitting level of the cation. 

Trivalent lanthanide cations (Ln^3+^) are prevalent in chelates. Ln^3+^ possess a 4f^n^ subshell, wherein n values vary from n = 1 (in Ce^3+^) to n = 14 (in Lu^3+^). Strongly attracted to the positively charged nucleus and shielded from any external forces by the filled 5s and 5p orbitals, the 4f electrons are not susceptible to photoexcitation. This phenomenon is reflected in the low extinction coefficients, sharp absorption and emission bands and long-lived luminescence. Upon coordination, the energy is absorbed by the ligand and transferred to the cation, which ultimately leads to emission. Thus, the excitation and emission wavelengths are characteristic of the ligand and the lanthanide ion, respectively. The energy-transfer process can be intramolecular or intermolecular [82]. 

**Figure 6:**
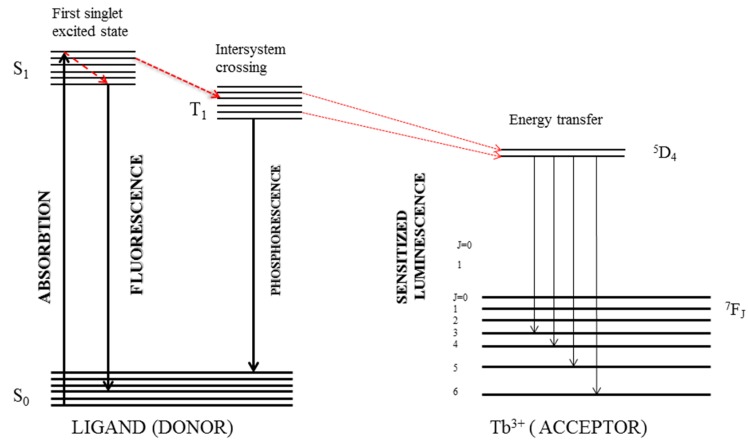
Intramolecular energy transfer for Tb^3+^, with permission from [81].

The intramolecular process requires the formation of a chelate. The light energy absorbed by the ligand in its ground state (S_0_) leads to the generation of an excited single state (S_1_). Energy is then transferred through a process called intersystem crossing to an excited triple state (T_1_), from which the energy can be transferred to the 4f electrons of the lanthanide ion. Thus, these electrons become excited, and upon returning to their ground state, emit radiation at characteristic wavelengths. The energy transfer depends on the ligand structure and the energy level of its triple state, which has to be higher than but close to the resonance level of the lanthanide ion. The mechanism is shown for Tb^3+^ in Figure 6 [81]. Moreover, there are other non-radiative and radiative mechanisms that can compete with this particular energy transfer and luminescence, such as fluorescence, phosphorescence and non-radiative relaxation. In order to minimize the effects of these processes, synergistic agents and micellar solutions are normally used. Tb^3+^ and Eu^3+^ have been preponderantly used for analytical purposes, due to their longer decay times and intense emission bands at 545 nm and 612 nm, respectively [80]. 

The intermolecular process implies no chelate formation; the energy is transferred from the triple state of an organic compound, such as an aromatic aldehyde or ketone, to the 4f electrons of the lanthanide ion. The process is diffusion controlled, induced through collisional quenching and takes place after the encounter between the donor and the acceptor [82].

These processes present a series of characteristics, such as large Stokes shifts and narrow emission bands, which render them suitable for analytical purposes, allow for low sample blanks and minimize overlapping. All of the above-mentioned characteristics result in high sensitivity and selectivity. Moreover, long-lived excited states enable the implementation of time-resolution detection techniques [83].

The luminescent properties of a lanthanide–quinolone complex are structure-related. Upon complexation, the conjugate plane increases and the structure becomes more rigid. A higher stability of the complex results in a smaller energy loss and a more efficient energy transfer, which increases the luminous efficiency of Ln^3+^. Small variations in quinolone rings and substituent groups greatly influence these phenomena. For instance, the ether group (ofloxacin, gatifloxacin) or thioether group (rufloxacin) in the R_1_ position (see Figure 1c) results in p–π conjugation with the quinolone ring, creating an electron pushing effect which reduces the electron density in the carbonyl and carboxyl groups, diminishing the coordination ability of the quinolone. This results in weak energy transfer and low luminescence intensity. In the case of sparfloxacin, the –NH_2_ group in the R_5_ (see Figure 1c) position forms an intramolecular hydrogen bond with the carbonyl group, causing a non-planar twist of the pyridine ring, which in turn reduces the π-bond’s degrees of freedom. This results in quenched luminescence of the quinolone and its complexes [78].

In order to minimize the ligand deactivating processes and to optimize chelate formation, different methods have been used: use of synergistic agents; changes in pH, surfactants, columinescence or heavy atoms or use of a solid phase [81]. 

Lanthanide cations are known to have high coordination numbers in the range of 6–12. In cases where only two quinolone molecules are bound to the central ion, other ligands, such as 1,4,7,10-tetraazacyclododecane-1,4,7-triacetic acid (DO3A) [84] or tri-n-octylphosphine oxide (TOPO), have been used. This strategy proved to be necessary in order to avoid filling the coordination sphere with solvent molecules, represented in most cases by water, which will result in non-radiative deactivations. These ligands create a sheath around the complex, assuring desolvation [85], or can fill in the coordination sphere of the lanthanide ion [86]. Moreover, the removal of oxygen atoms from the coordination sphere enhances luminescence [87,88,89].

The zwitterionic nature of fluoroquinolones renders their structures and abilities to form metal complexes to be greatly affected by pH. Thus, the increase of the pH values leads to an increased rate of complex formation, but also favors the rate of hydrolysis of the lanthanide cation. The most frequently used pH values lie in the range of 5–9 (see Table 4 and citations therein). The systems using chemiluminescence imply the use of acidic pH for the redox reaction. 

There is a drastic increase in fluorescence in microheterogeneous organized media rather than homogenous solutions [90,91]; micellar solutions obtained with anionic surfactants have the role of bringing the reactants together by trapping the energy donor inside the micelle and binding the lanthanide ion to its surface [85], enhancing the stability of the complexes and shielding them from quenchers [91]. The same effect can be obtained by using coordination polymer nanoparticles (CPNPs) [92], coordination polymer nanosheets (CPNSs) [93] or nanoparticles (NPs) [84,94,95,96,97,98,99].

The source of the electromagnetic radiation necessary to obtain the excited state of the ligand is either the Xe lamp of the spectrofluorometer, or a chemical species in an excited state, such as SO_2_^*^ obtained through a chemical reaction (chemiluminiscence). Numerous methods have been developed for the determination of quinolones [100,101,102,103,104,105,106,107,108,109], based on the formation of an excited and unstable chemical species in a redox system. This chemical species emits radiation capable of being absorbed by the fluoroquinolone. Several notable systems and mechanisms have been described as follows: the systems are formed from an oxidant, such as Ce^4+^ or KMnO_4_, in an acidic medium, and a sulfur containing a reducing agent, such as S_2_O_4_^2−^, S_2_O_3_^2−^ or Na_2_SO_3_, which will eventually generate the excited SO_2_^*^ species. This species emits at a wavelength around 350nm at which the fluoroquinolone is able to absorb [101]. Some interesting examples are given below:

a.FQ-Tb^3+^-Ce^4+^-SO_3_^−^-H_2_SO_4_ system:

Ce^4+^ + HSO_3_^-^ → Ce^3+^ + HSO_3_^*^ 2HSO_3_^*^ → S_2_O_6_^2-^ + 2H^+^ S_2_O_6_^2−^ → SO_4_^2-^ + SO_2_^*^SO_2_^*^ + Ln(FQ)_2_^3+^ → SO_2_ + Ln(FQ^*^)_2_^3+^ Ln(FQ^*^)_2_^3+^ → Ln^*^(FQ)_2_^3+^ Ln^*^(FQ)_2_^3+^ → Ln(FQ)_2_^3+^ + hν, FQ = levofloxacin, moxifloxacin, trovafloxacin [110];b.FQ-Ce^4+^-S_2_O_4_^2−^-HNO_3_ system:

S_2_O_3_^2^^−^*/*S_2_O_4_^2^^−^ + H^+^ → HSO_3_^−^ + H_2_O Ce^4+^ + HSO_3_^−^ → HSO_3_^∗^ + Ce^3^^+^ 2HSO_3_^∗^ → S_2_O_6_^2^^−^ + 2H^+^ S_2_O_6_^2^^−^ → SO_4_^2^^−^ + SO_2_^∗^SO_2_^∗^ → SO_2_ + *hν*
Ce^4+^ +FQ + H^+^ → Ce^3^^+^ + FQ oxide + H_2_O SO_2_^∗^ +FQ oxide → FQ oxide^∗^ + SO_2_ FQ oxide^∗^ → FQ oxide + *hν*, FQ = norfloxacin [109];c.Ln^+3^-FQ-Ce^4+^-S_2_O_4_^2−^ system:

S_2_O_4_^2^^−^*/*S_2_O_3_^2^^−^ + H^+^→ HSO_3_^−^ Ce^4^^+^ + HSO_3_^−^→ HSO_3_^∗^ + Ce^3^^+^ 2HSO_3_^∗^ → S_2_O_6_^2^^−^ + 2H^+^ S_2_O_6_^2^^−^ → SO_4_^2^^−^ + SO_2_^∗^SO_2_^*^ + [Ln–FQ]^3+^→SO_2_ + [Ln–FQ^*^]^3+^
[Ln–FQ^∗^]^3^^+^→ [Ln^∗^–FQ]^3^^+^
[Ln^∗^–FQ]^3^^+^→ [Ln–FQ]^3^^+^+*hν*,
FQ = gatifloxacin, enoxacin, Ln = Eu^3+^, Dy^3+^ [109].

It is well-known that the evaluation of the performance of these methods is concerned with the accurate response to target fluoroquinolones in the presence of other coexisting antibiotics and chemotherapeutics. Thus, the selectivity of the methods has been tested based on possible interferences with other various chemical species; e.g., metal ions, penicillins, cephalosporins, amino-glycosides, macrolides, ionophores and sulfonamides [117,122,138,163]. For instance, selective and sensitive detection in serum and urine has been reported for a fleroxacin “turn-on” sensing device; namely, the Eu^3+^-Pb-MOF system, where MOF = metal organic framework, [(Me)_4_N]_2_[Pb_6_K_6_(m-BDC)_9_(OH)_2_]·H_2_O, where 1,3-H_2_BDC = 1,3-benzenedicarboxylic acid), λ_em_ = 612 nm (λ_exc_ = 377 nm). The detection limit for fleroxacin is low (118 nM) and the sensor possesses high selectivity for fleroxacin, as opposed to the other seven fluoroquinolones tested; high antidisturbance ability to other chemicals in human serum and urine has also been reported. The probe was also proven to be stable in aqueous solutions of pH values ranging from 4.0 to 8.3 [134]. 

### 4.2. Quantitative Determination of Lanthanide and Miscellaneous Ions

Norfloxacin was used to determine erbium, holmium and neodymium in the presence of cetylpiridinium chloride by recording the second derivative spectrum of absorbance. The effect of interfering cations was also tested [164]. 

The Eu^3+^-La^3+^-gatifloxacin-SDBS system has been found appropriate to determine trace amounts of europium. Optimum conditions were tested and applied. In order to explain the luminescence enhancement mechanism, the group recorded UV-absorption spectra, determined the surface tension and the microviscosity of the aqueous system and concluded that the quaternary complex was formed in a highly viscous and hydrophobic medium with no water molecules to quench the luminescence through radiationless vibrations. Therefore, the complex was able to fully transform the absorbed energy into luminescence [165].

Other cations can also be determined using lanthanide–quinolone complexes. For instance, a method that makes use of the quenching effect of Cu^2+^ on the luminescence of a Tb^3+^-quinolone complex has been reported for the quantitative determination of Cu^2+^ ions from tap water samples. This method enabled the detection of Cu^2+^ ions in a nanomolar range. Further studies show that the quenching mechanism is a static one, the Cu^2+^ ions being able to bind to the nitrogen atoms of the thiazolidine ring, thereby coordinating it and forming a complex that is not fluorescent in ground state [166]. 

Another use for the phenomenon of complex formation is the extraction of a metal from a medium in the presence of different cations and anions. The complex formed by nalidixic acid with Sc^3+^ was used for the extraction of the latter from dichloromethane, in the presence of various lanthanides or transition metal cations. After separation, the metal concentration of each phase was determined radiometrically. The results of this study in terms of selectivity, recycling ability and loading capacity were reported to be excellent under the stated optimized conditions [167]. 

Quantitative determination of anions by similar methods has also been reported. The luminescence quenching effect of the phosphate anion on the Tb^3+^-4-hydroxy-1-methyl-2-oxo-1,2-dihydro-quinoline-3-carboxylic acid-ethyl-[1,3,4]thiadiazol-2-yl) amide was used in order to determine the former. The system was analyzed in aqueous solution, at a pH value of 7.4, using λ_ex_ = 320 nm and λ_em_ = 548 nm. This method allows for the determination of phosphate anions in the micromolar range. Further tests indicate that the quenching mechanism is a static one [168]. 

The data describing these methods have been reported in Table 5.

### 4.3. Determination of Nucleic Acids

Fluorescent methods for the determination of DNA and RNA have been extensively used [169,170], despite of their downside; namely, the toxic effects of the commonly used dyes, such as ethidium bromide (EB), Hoechst 33258, YOYO, TOTO and phosphine 3R, on humans and the environment [171].

The fluorescence of the quinolone–lanthanide complexes is not only used for the determination of quinolones, but also for organic compounds of biological interest, such as nucleic acids. Highly sought traits of these complexes for the determination of nucleic acids are listed as: high luminescence, large quantum yield, high kinetic stability and good water solubility [172].

Furthermore, several terbium complexes have been reported as suitable candidates for the quantitation of DNA. The structure of the ligand was optimized (including the addition of sidechains containing a quaternary ammonium group) in order to enhance the solubility of the complexes in aqueous buffers, and to enable DNA intercalative binding. DNA binding was found to occur by means of hydrogen bonds formed between the non-divided electron pairs of the nitrogen atoms in DNA and the quaternary ammonium group. The most efficient ligand was found to be *N*-[2-(diethylamino)ethyl]-4-hydroxy-2-oxo-1-propyl-1,2-dihydro-3-quinolinecarboxamide. Interestingly, an increase of up to 10-fold in luminescence of its complex with Tb^3+^ was observed upon titration with DNA. The method displays comparable sensitivity to the EB method. Moreover, in contrast to other techniques reported for DNA determination that employ lanthanide complexes, the addition of surfactants or other luminescence enhancers was found to be unnecessary [172].

A Tb^3+^-prulifloxacin complex was used to determine herring sperm DNA (hsDNA). The method, using a 2:1 ligand to ion ratio and pH = 6.3, proved to be more stable, less toxic to the environment and highly sensitive. The limit of detection obtained for DNA was 2.1 × 10^−9^ g/L [172]. The intercalative binding of the complex to the DNA molecule was proven using Ru(bpy)_2_(dppz)^2+^, a known DNA intercalator [173]. 

Calf, herring and salmon sperm DNA were determined using the complex of Eu^3+^ with gatifloxacin in the ratio 1:3.1, at pH = 6.5 buffered with hexamethylenamine-HCl solution. Upon adding the DNA sample, a 6-fold increase in the fluorescence was observed. The influence of the ionic strength on the fluorescence of the system hinted at an electrostatic interaction between the complex and DNA. Interestingly, the fluorescence enhancement mechanism was explained as follows: the coordination number of Eu^3+^ is eight; therefore, three gatifloxacin molecules and two water molecules are needed to saturate it; the water molecules partially absorb the energy transferred from the excited state of gatifloxacin to Eu^3+^, causing a drop in the fluorescence signal. The DNA molecule can displace the H_2_O molecules and bind Eu^3+^ either by complexation (via O and N) or by means of electrostatic interactions with the phosphate groups. Thus, the loss of the water molecules, and implicitly the decrease in energy loss through the O-H vibration, results in a stronger fluorescent signal [174].

In a similar manner, the complex of difloxacin with Tb^3+^ was used for the determination of both single-stranded and double-stranded DNA from calf thymus, herring and salmon sperm. The binding propensity towards double-stranded DNA was higher than towards single-stranded DNA. The influence of the ionic strength of the solution upon the emission spectra and EB displacement assay were employed in order to investigate the mechanism of DNA binding. The results suggest that the Tb-difloxacin complex binds DNA via electrostatic interactions with the phosphate groups and groove binding [175].

Furthermore, fluorescent properties can be enhanced by attaching the lanthanide complex to an oligonucleotide [176,177]. Oligonucleotide probes are single-stranded nucleic acid fragments that are modified to have high specificities and affinities for certain targets, such as nucleic acids, proteins, small molecules and ions. They can be divided into two categories: hybridization probes which are based on the formation of complementary base pairs and aptamer probes which exploit selective recognition of non-nucleic acid analytes by binding to them as to a 3D receptor. Binding of the analyte to the bioreceptor can generate an analytical signal using fluorescent groups that are covalently bound to the predefined oligonucleotide. The advantages of using fluorescent labels are their high specificity and their ability to be used in assays that interpret the fluorescence signal in various ways: quenching or enhancement, anisotropy, lifetime, resonance energy transfer and the excimer–monomer light switch [178]. 

Luminescent terbium SiO_2_ nanoparticles covered with a lanthanide complex were used for DNA determination. This complex consists of a quinolone-based dye (Carbostyril 124, Cs124), one of the most efficient energy-transfer donors for terbium and europium, and a polyaminocarboxylate-based chelator which has excellent water solubility and high lanthanide binding affinity. The complex was then hybridized by attaching it to an oligonucleotide. The detection sensitivity of this method proved to be more than 100-fold higher in comparison to the fluorescein isothiocyanate method [179].

In a similar manner, diethylenetriaminepentaacetic acid (DTPA) was attached to 7-amino quinolones in order to obtain DTPA-cs124 derivatives (DTPA-cs214-CF_3_-NCS and DTPA-cs124-NCS); Tb^3+^, Eu^3+^, Dy^3+^ and Sm^3+^ cations were used for complexation. The probes were hybridized with oligonucleotides complementary to the DNA sequence of interest. The detection limit is reported to be 0.5–1 × 10^−12^ mol/L [177]. 

The data describing these methods have been displayed in Table 6.

### 4.4. Other Applications

Fluorescence properties can be used to measure the stability constants of metal–fluoroquinolone complexes, and to elucidate, to some extent, the structures of these complexes. The difference in fluorescence spectra between the free acidic ligand-conjugated form and/or the complex ligand can give information about ligand protonation and the stability of the complex.

Spectrofluorimetric titrations are carried out by additions of known quantities of the metal ion to the buffered solutions of the ligand, while registering the fluorescence maximum increase, decrease or shift, depending on the metal and the complex formed [181]. Luminescence enhancement due to complex formation can be briefly explained as follows: (a) the extended π–π ring conjugation in the complex results in fluorescent enhancement; (b) free rotation of the carboxylate or the hydroxyl of the carboxylate group is inhibited, a phenomenon which decreases the non-radiative energy loss; (c) the short distance between the metal ion and the ligand enhances the energy transfer and the blue-shift of the maximal fluorescence wavelength.

**Figure 7:**
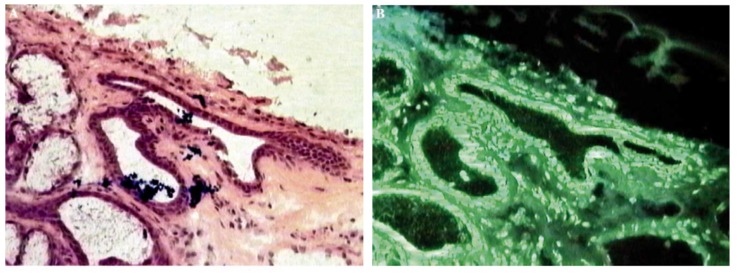
(**a**) Hematoxylin and eosin coloring technique. (**b**) Coloring with quinolone–lanthanide complexes, with permission from [182].

Furthermore, Tb^3+^ and Eu^3+^ complexes with ciprofloxacin and ofloxacin, respectively, were used to stain sections of normal and malignant tissues from oral cavities and stomachs (Figure 7). Transformed-infrared (FT-IR) and fluorescence spectroscopy studies revealed that ATP coordinates to Eu^3+^ and Tb^3+^, releasing free ciprofloxacin and ofloxacin; other interactions include hydrogen bonds between the chelates and the biomolecules, and coulomb forces between the negatively charged carboxyl groups of the biomolecules and NH_2_^+^ groups of the quinolones. Moreover, it was observed that the complex is absorbed differently in distinct tissues, which helps to differentiate the tissues. Compared to the traditional hematoxylin and eosin coloring technique, the fluorescence staining method is very fast; the samples can be preserved at room temperature for at least two years; on the downside, this method needs a more expensive fluorescent optical microscope compared to the optical microscope used for the traditional method [182].

An Eu^3+^-ofloxacin complex, able to change its color depending on the pH variation of the medium, and which is completely water-soluble, was incorporated into a polyvinyl alcohol matrix, making possible the development of two lanthanide luminescent hydrogels (rod-shaped and film) [183]. 

The complexes formed between an ofloxacin derivative with Pr^3+^ and Nd^3+^ were used to evaluate the interaction with bovine serum albumin (BSA) based on the quenching of its luminescence at 341 nm. Experiments proved that the quenching was a static process due to hydrophobic and electrostatic interactions which could induce conformational changes to BSA [55].

The interaction between Ce^3+^ ions and carbonyl group was used in order to synthesize some quinolone antibiotic norfloxacin analogues through a new pathway [184]. 

Interestingly, expired fluoroquinolone tablets can be reused as Tb^3+^ or Eu^3+^ complexes. These metal complexes were ultimately incorporated into homogenous films for greenhouses and used for converting UV light (366 nm) into visible light (615 nm) that can be used by plants in the photosynthesis process [185].

## 5. Conclusions

The field of lanthanide–quinolone complexes aims to develop new candidates that are more active than the free ligand, which could be useful in the fight against bacterial or tumoral resistance, and has applications in analytical determinations and tissue imaging. Despite the progress made over the last decade in developing new biologically relevant quinolone–lanthanide metal complexes, in the past two years a decrease in the number of publications has been observed. With little explored to date, metal complexes with mixed ligands (e.g., 1,10-phenantroline, 2,2′-dipyridyl) and the nanocomplexes show great promise as a future area of research, based on their biological activities.

On the other hand, the phenomenon of complex formation and the luminescent properties of the resultant compounds are still in focus, the main application being the quantitative determinations of quinolones based on their reactions with lanthanide ions. Likewise, quantitative determination of metal ions based on their reactions with lanthanides, determinations of nucleic acids and staining sections of normal and malignant tissues for fluorescence spectroscopy, have also been investigated.

## Figures and Tables

**Table 1:** Methods for the synthesis of quinolone complexes with lanthanide ions.

Ligand	Complex	Material/Ligand:Ln Molar Ratio/pH Adjustment	Mixing Mode	Heating/Cooling	Ref
**Pipemidic acid (PPA)**	[M(PPA)_4_Cl]Cl_2_ where M = La^3+^; [M(PPA)_4_]Cl_3_ where M = Ce^3+^, Pr^3+^, Nd^3+^, Sm^3+^, Tb^3+^, Dy^3+^, Y^3+^.	-ligand salt dissolved in ethanol mixed with PPA dissolved in acetic acid; -4:1; -no pH adjustment.		-refluxed on water bath 3–4 h; -cooling to room temperature.	[39]
**Nalidixic Acid (NAL)**	[Ln(NAL)_3_]⋅nH_2_O n = 5 for La^3+^ n = 6 for Ce^3+^, Pr^3+^, Sm^3+^.	-aqueous solution of nalidixic salt with Na^+^, pH = 8.5; -aqueous solution of LnCl_3_, -pH = 5.	-nalidixate solution added slowly and under continuous stirring.	-heated until boiling, then kept in refrigerator 12 h; -deionized water added and repeated.	[40]
**Ciprofloxacin (CPX)**	[Ln(CPX)_2_]Cl⋅nH_2_O, n = 7–9, Ln = Pr^3+^, Nd^3+^, Sm^3+^, Eu^3+^, Gd^3+^, Tb^3+^, Dy^3+^, Ho^3+^, Er^3+^, Tm^3+^, Yb^3+^.	-ciprofloxacin HCl, lanthanide oxides; -2:1.	-CPFX dissolved in HCl solution and pyridine; -Ln(III) oxides dissolved in conc. HCl, heated to dryness and dissolved in water; -lanthanide solution dropped into CPFX, continuous stirring (2 h–5 days).		[41]
	[Ce(CPX)_2_(H_2_O)_4_]Cl⋅ (H_2_O)_3.25_⋅(C_2_H_5_OH)_0.25_	-ciprofloxacin HCl, CeCl_3_⋅7H_2_O; -2:1.	-lanthanide dissolved in water, added to CPFX solution.	-stirring for 7 days at room temp; -20 mL ethanol added, left standing at room temp.	[41]
	[Eu(CPXH)(CPX)(H_2_O)_4_]Cl_2_⋅ 4.55H_2_O	-EuCl_3_⋅6H_2_O, ciprofloxacin HCl hydrate, 1,3-bis (dimethylamino)- 2 propranolol; -2:1.	-solvothermal method: substances in mixture of water and ethanol -placed in stainless steel autoclave.	-heating at 150 °C for 48 h, cooling; -isothermal evaporation in the air.	[42]
	[La(H_2_O)_4_(CPX)_2_]Cl	-LaCl_3_, ciprofloxacin; -2:1.	-both substances suspended in methanol, stirred for 10h.	-reflux 8 h.	[43]
**Enrofloxacin (EF)**	[La_2_(EF)_6_(H_2_O)_2_]⋅14H_2_O, [Sm_2_(EF)_6_(H_2_O)_2_]⋅14H_2_O	-La_2_O_3_, Sm_2_O_3_ enrofloxacin, distilled water; -2:1.	-components sealed in 25ml Teflon- lined stainless vessel.	-heated at 435 K, 3 days; -cooled at room temp.	[44]
**Gatifloxacin (GFLX)**	[Ln(GFLX)_3_Cl_3_]⋅2H_2_O, Where Ln = La^3+^, Nd^3+^, Eu^3+^, Tb^3+^.	-GFLX, LnCl_3_⋅6H_2_O; -3:1.	-HGA dissolved in acetone added dropwise into solution of LnCl_3_⋅6H_2_O dissolved in ethanol.	-stirred and refluxed for 2 h.	[45]
	[Eu(GFLX)_3_⋅2H_2_O]	-gatifloxacin, EuCl_3_⋅6H_2_O; -3:1; -pH = 9 with ammonium hydroxide.	-gatifloxacin and Eu^3+^ salt dissolved in deionized water, while stirring; -yellow solution obtained;	-stirring 2 h at room temp.	[46]
**Gemifloxacin (GMFX)**	[La(GMFX)_2_(H_2_O)_2_]Cl_3_⋅3H_2_O [Ce(GMFX)_2_(H_2_O)_2_](SO_4_)_2_⋅2H_2_O.	-gemifloxacin, LaCl_3_⋅7H_2_O, Ce(SO_4_)_2_; -2:1.	-substances dissolved in acetone, metal salt solution added dropwise to the solution of gemifloxacin.	-stirring for 15 h at room temp.	[47]
	[La(GMFX)(phen)(H_2_O)_2_]Cl_3_⋅6H_2_O [Ce(GMFX)(phen)(H_2_O)_2_] (SO_4_)_2_⋅3H_2_O.	-gemifloxacin, 1,10- phenanthroline, LaCl_3_⋅7H_2_O, Ce(SO_4_)_2_; -GMFX: metal: phen = 1:1:1.	-mixing hot saturated solution on GMFX in acetone with phen and metal salt.	-reflux for 3 h.	[48]
	[Ce(GMFX)(Gly)(H_2_O)_2_]Cl_2_⋅ H_2_O	-gemifloxacin, glycine, CeCl_3_⋅7H_2_O; -GMFX: metal: Gly = 1:1:1.	-metal salt dissolved in ethanol added dropwise to a stirred solution of GMFX and Gly in ethanol;	-reflux and stirring 5–6 h at 100 °C in water bath;	[49]
**Levofloxacin (LEVO)**	[Ce(LEVO)_2_(H_2_O)_2_]SO_4_⋅5H_2_O	-levofloxacin, Ce(SO_4_)_2_; -2:1.	-metal salt added to magnetically stirred solution of LEVO and NaOH in methanol and acetone.	-stirring at room temp for 1 day.	[50]
**Moxifloxacin (MOXI)**	[Ce(MOXI)_2_](SO_4_)_2_⋅2H_2_O	-moxifloxacin, (NH_4_)_4_Ce(SO_4_)_4_⋅2H_2_O -2:1.	-ethanolic solution of metal salt added dropwise to a stirred ethanolic solution of moxifloxacin.	-24 h stirring at 50 °C on water bath.	[51]
**Norfloxacin (NOR)**	[N(CH_3_)_4_][Ln(NOR)_4_]⋅6H_2_O, where Ln = Nd^3+^, Sm^3+^, Ho^3+^; [N(CH_3_)_4_] = tetramethylammonium.	-norfloxacin, Ln(NO_3_)_3_⋅6H_2_O, imidazole; -3:1.	-norfloxacin, metallic salt and imidazole mixed with distilled water and sealed in a 25ml Teflon-lined stainless vessel.	-heating at 453 K for 3 days; -cooled slowly to room temp.	[52]
	[La(NOR)_3_]⋅3H_2_O, [Ce(NOR)_3_]⋅2H_2_O	-norfloxacin, CeCl_3_⋅7H_2_O, LaCl_3_⋅6H_2_O; -3:1; -basic pH with ammonia solution 5% *v*/*v*.	a) -NOR suspended in methanol mixed with metal salt suspended in distilled water; -solution basified by adding ammonia solution; b) complexes obtained as nanoparticles by the following transformations: metal chlorides→metal carbonates→nano-oxides→metal chlorides→above described method;	a) -reflux at 80–90 °C for 4–5 h; b) -reflux at 80–90 °C for 4–5 h.	[53]
	[La(NOR)_2_Cl_2_]Cl	-2:1.	-substances suspended in methanol.	-gently stirring for 10 h and reflux for 8 h.	[54]
**Ofloxacin (OFLO)**	[Pr(L-OFLO)(NO_3_)_2_(CH_3_OH)](NO_3_), [NdOFLO(NO_3_)_2_(CH_3_OH)](NO_3_), where L-OFLO = derivative obtained by esterification of ofloxacin.	-ofloxacin, NaOH, Pr(NO_3_)_3_⋅6H_2_O, Nd(NO_3_)_3_⋅6H_2_O; -ligand: NaOH: metal = 0.25: 0.25: 0.3.	-ligand (L) obtained by esterification of ofloxacin with ethanol, treatment with N_2_H_4_⋅H_2_O and refluxed with 2-hydroxybenz-aldehyde; -ligand and NaOH mixed in methanol; metal salt added.	-refluxed and stirred for 3 h; -cooling to room temperature.	[55]
	[Pr(L-OFLO)_2_(NO_3_)](NO_3_)_2_, where L-OFLO = ofloxacin derivative.	-ofloxacin, NaOH, Pr(NO_3_)_3_⋅6H_2_O; -ligand: NaOH: metal = 0.25: 0.25: 0.3.	-ligand (L) obtained by esterification of ofloxacin with ethanol, treatment with N_2_H_4_⋅H_2_O and refluxed with 2-hydroxybenz-aldehyde; -ligand and NaOH mixed in methanol; metal salt added.	-stirring 3 hat room temp;	[56]
**Sparfloxacin (SPAR)**	[La(SPAR)_2_NO_3_⋅H_2_O]⋅2H_2_O (1), [La(SPAR)(HL)NO_3_⋅H_2_O]⋅H_2_O (2), where L = DL-alanine.	-sparfoxacin, La(NO_3_)_3_⋅6H_2_O, DL-alanine; -sparfloxacin: metal = 2; 1; -sparfloxacin: DL-alanine: metal = 1: 1: 1.	-metal salt dissolved in hot water -ethanolic solution added to a hot ethanolic solution of sparfloxacin and DL-alanin.	-mixture stirred under reflux for 2 h; left to cool.	[57]

**Table 2:** Biological activity of quinolone complexes with lanthanide ions.

Ligand	Complex	Biological Activity Test	Results	Ref
**Pipemidic acid (PPA)**	[La(PPA)_4_Cl]Cl_2_	-antibacterial activity on *E. coli, S. aureus, P. aeruginosa* similar to PPA;	-antibacterial activity on *S. pneumoniae* much greater than PPA; -NO activity on *S. aureus*	[39]
	[M(PPA)_4_]Cl_3_ where M = Ce^3+^, Pr^3+^, Nd^3+^, Sm^3+^, Tb^3+^, Dy^3+^, Y^3+^.		-Pr, Sm, Y complexes have similar activity to PPA against *E. coli, S. aureus, B. subtilis, S. pneumoniae*; weaker activity on *P. aeruginosa*.	[39]
**Ciprofloxacin (CPX)**	[Er(CPX)_2_(H_2_O)_8_]Cl [Ce(CPX)_2_(H_2_O)_4_]Cl·(H_2_O)_3.25_(C_2_H_5_OH)_0.25_	-MIC determined by broth tube dilution method; *E. coli, P. aeruginosa, S. aureus*;	-for Ce, the activity is 2.5, 2.5,1.25 fold higher than CPX; -for Er, the activity is 2.5, 1.25 fold higher, resp. 3.0 fold lower than CPX.	[41]
	[La(H_2_O)_4_(CPX)_2_]Cl	-antibacterial activity against *E. coli* strains, through flat-filter paper method;	-the complex is less active than ciprofloxacin.	[43]
**Enrofloxacin (EF)**	[La_2_(EF)_6_(H_2_O)_2_]⋅14H_2_O [Sm_2_(EF)_6_(H_2_O)_2_]⋅14H_2_O	-antibacterial activity tested against *B. subtilis, S. aureus, E. coli* through the diffusimetric method;	-both complexes have bactericidal properties greater than the ligand; -the Sm^3+^ complex is more active than the La^3+^ complex.	[44]
**Gemifloxacin (GMFX)**	[La(GMFX)_2_(H_2_O)_2_]Cl_3_⋅3H_2_O [Ce(GMFX)_2_(H_2_O)_2_] (SO_4_)_2_⋅2H_2_O	-antibacterial activity tested against *S. aureus, B. subtilis, E. coli, P. aeruginosa*, by diffusimetric method; -antifungal activity tested against *C. albicans, A. awamori, Altenaria sp.* by diffusimetric method; -cytotoxic activity tested against human breast carcinoma cell line (MCF-7 cells), human colon carcinoma cell line (HCT-116 cells), through crystal violet colorimetric viability assay;	-the activity of the La^3+^ complex is comparable to gemifloxacin, but the one of the Ce^4+^ complex is slightly higher; -only the Ce^4+^ complex is active and only against *C. albicans*; -results were compared to the activity of doxorubicin; both complexes were found to be active on both cell lines, but have IC_50_ higher than doxorubicin; Ce^4+^ complex more active than La^3+^ complex against the breast carcinoma cell line; La^3+^ complex more active than the Ce^4+^ complex against the colon carcinoma cell line.	[47]
	[La(GMFX)(phen)(H_2_O)_2_] Cl_3_⋅6H_2_O [Ce(GMFX)(phen)(H_2_O)_2_] (SO_4_)_2_⋅3H_2_O	-antibacterial activity against *S. aureus, B. subtilis, E. coli, P. aeruginosa*, by diffusimetric method; -antifungal activity tested against *C. albicans, A. awamori, Alternaria sp.* by diffusimetric method; -cytotoxic activity tested against human breast carcinoma cell line (MCF-7 cells) and human colon carcinoma cell line (HCT-116 cells) by crystal violet staining viability assay; doxorubicin used as positive control;	-activity against *S. aureus* is comparable to GMFX; higher activities against the others; -comparable activity against *C. albicans* to GFX; no activity against other fungi strains; -complexes show cytotoxic activity, but lower than GMFX; phen also shows cytotoxic activity, but higher than GMFX.	[48]
	[Ce(GMFX)(Gly)(H_2_O)_2_] Cl_2_⋅H_2_O	-antibacterial activity tested against *Xanthomonas campestris, Bacillus megeterium, E. coli, Clavibacter michiganesis;* -antifungal activity tested against phytopathogenic fungi: *Rhizoctonia solani, Sclerotinia sclerotium, Aspergillus niger, Botrytis cinerea, Penicillium digitatum*; -antioxidant activity tested through DPPH and ABTS methods;	-GMFX and complex proved to be active against all strains, the weakest activity being against *E. coli* and the highest against *C. michiganensis*; -complex shows lower activity than GMFX against all strains; -complex activity comparable to that of GMFX.	[49]
**Levofloxacin (LEVO)**	[Ce(LEVO)_2_(H_2_O)_2_]SO_4_⋅ 5H_2_O	-antibacterial activity tested against *S. aureus, B. subtilis, B. otitidis, E. coli, P. aeruginosa, K. oxytoca*, by cup-diffusion technique; -antifungal activity tested against *A. flaurus, A. fumigatus*, using the disc diffusion sensitivity method;	-the Ce^4+^ proves to be more active on *B. subtilis* and *B. otitidis*; -no antifungal activity noted;	[50]
**Moxifloxacin (MOXI)**	[Ce(MOXI)_2_](SO_4_)_2_⋅2H_2_O	-antibacterial activity tested against *S. aureus, B. subtilis, B. otitidis, E. coli, P. aeruginosa, K. oxytoca* by cup-diffusion method;	-the complex shows similar activity against *E.coli;* -no activity against *P. aeruginosa* and *K. oxytoca*; -higher activity against *B. subtilis, B. otitidis* and *S. aureus*.	[51]
**Norfloxacin (NOR)**	[La(NOR)_3_]⋅3H_2_O [Ce(NOR)_3_]⋅2H_2_O	-antibacterial activities tested using modified Kirby-Bauer disk diffusion method, against *S. aureus, B. subtilis, E. coli, P. aeruginosa, C. albicans, A. flavus*; positive controls used: tetracycline and amphotericin;	-complexes in nanoparticle form displayed greater activities than those in normal- particle form, but lower than the positive controls; -La^3+^ nanocomplex is the most active.	[53]
**Ofloxacin (OFLO)**	[Pr(L-OFLO)(NO_3_)_2_(CH_3_OH)] (NO_3_) [Nd(L-OFLO)(NO_3_)_2_(CH_3_OH)] (NO_3_), where L-OFLO = ofloxacin derivative.	-antioxidant activity tested through hydroxyl radical scavenging activity through the Fenton reaction;	-complexes show better activity than the ligand.	[55,58]
**Sparfloxacin (SPAR)**	[La(SPAR)_2_NO_3_⋅H_2_O]⋅2H_2_O (1) [La(SPAR)(HL)NO_3_⋅H_2_O]⋅ H_2_O (2), where L = DL-alanin.	-antibacterial activity tested against *S. aureus, E. coli* using modified Kirby-Bauer disc diffusion method; tetracycline used as control; -antifungal activity tested against *A. flavus, C. albicans* using modified Kirby-Bauer disc diffusion method; amphotericin B used as control;	-the complexes show the same activity as the free ligand, which is higher than the control, against both bacteria; -complexes and free ligand showed no antifungal activity.	[57]

**Table 3:** Calf-thymus (CT-DNA) and bovine serum albumin (BSA) binding tests for quinolone complexes with lanthanide ions.

Ligand	Complex	Binding of Bovine Serum Albumin/CT-DNA	Results	Ref
**Ciprofloxacin (CPX)**	[La(H_2_O)_4_(CPX)_2_]Cl	-CT-DNA binding properties investigated through UV-VIS spectroscopy and fluorescence quenching methods;	-binding ability of CPX and complex is the highest in basic medium and the lowest in acidic medium.	[43]
**Enrofloxacin (CF)**	[La_2_(EF)_6_(H_2_O)_2_]⋅14H_2_O [Sm_2_(EF)_6_(H_2_O)_2_]⋅14H_2_O	-BSA binding properties investigated through UV-VIS spectroscopy and fluorescence quenching methods;	-both complexes have the ability to quench the fluorescence of BSA, the Sm^3+^ complex more than the La^3+^; -the mechanism is mainly a static one.	[44]
**Levofloxacin (LEVO)**	[Ce(LEVO)_2_(H_2_O)_2_]SO_4_⋅5H_2_O	-CT-DNA degradation by testing electrophoretic mobility;	-the complex degrades the DNA completely compared to the metal salt.	[50]
**Norfloxacin (NOR)**	[N(CH_3_)_4_][Ln(NOR)_4_]⋅ 6H_2_O, where Ln = Nd^3+^, Sm^3+^, Ho^3+^;	-CT-DNA binding investigated by UV absorbance of complex in the presence of increasing amount of CT-DNA and by emission spectra of EB-DNA;	-the complex binds to CT-DNA stronger than norfloxacin; -interaction with BSA tested by measuring fluorescence quenching spectra; -complexes have strong binding ability; -the quenching process is a static one.	[52]
	[La(NOR)_2_Cl_2_]Cl	-binding to CT-DNA, using UV-VIS absorption spectroscopy and time-resolved fluorescence spectroscopy;	-the complex shows moderate interaction with CT-DNA, by partial or non-intercalative binding modes.	[54]
**Ofloxacin (OFLO)**	[Pr(L-OFLO)(NO_3_)_2_(CH_3_OH)] (NO_3_) [Nd(L-OFLO)(NO_3_)_2_(CH_3_OH)] (NO_3_), where L-OFLO = ofloxacin derivative.	-binding to BSA using fluorescence quenching and UV-VIS spectroscopy; -DNA binding tested through viscosity measurement, electronic absorption titration, ethidium bromide (EB) displacement experiments, CD spectra and cyclic voltammetry experiments; -DNA cleavage tested by electrophoresis;	-tests confirm the intercalative binding mode of the complexes; interaction between complexes and DNA is stronger than of the free ligand; -the complexes show a concentration dependent activity through hydrolytic pathways.	[55,58]
	[Pr(L-OFLO)_2_(NO_3_)](NO_3_)_2_, where L-OFLO = ligand obtained from ofloxacin	-BSA binding tested through fluorescence quenching experiments; -CT-DNA binding tested through UV spectroscopy, UV titration experiments, competitive studies with EB, iodide quenching; -DNA cleavage tested through electrophoresis	-the complex binds to BSA with high affinity which induces a conformational change of BSA; -the complex binds to DNA more strongly than the free ligand; -the complex shows such activity.	[56]

**Table 4:** Methods for quantitative determination of quinolones based on complexation with lanthanides.

Quinolone	Method (System, Optimum pH, Optimum Buffer Conditions)	λ_exitation_/λ_emission_	Limit of Detection/Limit ofQuantification (LOD/LOQ)	Matrix	Ref
**Balofloxacin (BLFX)**	BLFX-Eu^3+^-SDBS system; pH = 7.5-tris HCl buffer;	335 nm/618 nm	LOD = 5 nM	Serum and urine samples	[111]
	BLFX-Eu^3+^-SDBS system; pH = 7.5-tris HCl buffer;	335 nm/618 nm	LOD = 1.3 nM	Bile Acid	[112]
	BLFX-Eu^3+^-KBrO_3_-Na_2_S_2_O_4_-SDBS chemiluminescence system	CL excitation/593 nm, 617 nm	LOD = 0.069 nM	Pharma-ceutical formulations and biological fluids	[113]
	BLFX-Eu^3+^-Y^3+^-SDBS system	330 nm/618 nm	LOD = 0.83 nM	Pharma-ceutical formulations, human serum, urine	[114]
**Ciprofloxacin (CPX)**	CPX-Tb^.+3^-TOPO system pH = 5.5-acetate buffer; micellar solution of CPCl	333 nm/546 nm	LOD = 1.2 nM	Serum samples	[85]
	CPX-Tb^3+^-SLS system; pH = 6- acetic acid- sodium acetate buffer;	300-325 nm / 549nm	LOD = 30–150 mol/kg	Chicken and trout muscle sample	[115]
	CPX-Ad/Tb^3+^ CPNP nanoparticles; pH = 7.5-HEPES buffer;	288 nm/545 nm	LOD = 60 nM	Aqueous solution, urine, tablets	[92]
	CPX-Tb^3+^-DO3A NPs; pH = 7.4-HEPES buffer;	278 nm/542 nm	LOD = 9 nM	Urine	[84]
	CPX-Tb^3+^-TOPO system; pH = 6-piperazine andimidazole buffer;	320 nm/545 nm	LOD = 100 nM	LC eluent	[116]
	CPX-Tb^3+^ system; pH = 5.7-potassium hydrogen phthalate buffer; (Additionally applied for: enrofloxacin and flumequine)	337 nm/545 nm	LOD = 9.6 nM	Water samples	[117]
	CPX-Tb^3+^-SDS-Na_2_SO_3_ system; pH = 6.5-acetate buffer;	271 nm/545 nm;	LOD = 1810.7 nM	Serum samples	[108]
	CPX-Tb^3+^-CTAB system; pH = 7.3-Tris buffer; in the presence of tetracycline.	284 nm/545 nm	LOD = 27162 nM	Serum and urine samples	[118]
	CPX-Tb^3+^ -SDS system; pH = 5.7-sodium acetate buffer;	278 nm/545 nm.	LOD = 422.5 nM; LOQ = 1508.9 nM.	Milk samples	[86]
	CPX-Tb^3+^-Ce^IV^–SO_3_^2-^ chemiluminescence system	CL excitation/ 490 nm, 545 nm, 585 nm 620 nm	LOD = 0.31 nM	Capsules, human serum and urine samples	[119]
	CPX- [Tb(bpy)_2_]^3+^–K_2_S_2_O_8_; pH = 5.5- acetic acid– sodium acetate buffer	ECL excitation/ 490 nm, 545 nm, 585 nm 620 nm	LOD = 1.4 nM	Pharma-ceutical tablets	[120]
	CPX- Tb^3+^-calf thymus DNA; pH = 5.5- acetic acid– sodium acetate buffer	272 nm/545 nm	LOD = 37.9 nM	eye-ear pharma-ceutical dosage forms	[121]
	CPX-Eu^3+^-phen system; pH = 9-acetate-ammonia buffer; micellar solution of DDBS.	330 nm/615 nm	LOD = 230 nM	Pharma-ceutical tablets, blood serum	[91]
	CPX-Eu^3+^-GMP NPs; pH = 7.4-HEPES buffer;	276 nm/615 nm	LOD = 780 nM	Pharma-ceutical tablets	[94]
	CPX-Eu^3+^ system in acetonitrile; pH = 6-borate buffer;	365 nm/615 nm	LOD = 15 nM;LOQ = 45 nM	Pharma-ceutical tablets, serum	[122]
	CPX-Eu^3+^-Ag NPs; pH = 9.4-tris-HCl buffer	373 nm/614 nm	LOD = 0.057 nM	Pharma-ceutical tablets, serum	[123]
	CPX-Eu^3+^ functionalized Ga(OH)(btec)·0.5H_2_O, where H_4_btec = 1,2,4,5-benzenetetra-carboxylic acid	370 nm/614 nm	LOD = 7243 nM	Urine samples	[124]
**Danofloxacin (DAN)**	DAN-Tb^3+^-SDS-Na_2_SO_3_ system; pH = 6.5-acetate buffer;	271 nm/545 nm;	LOD = 1810.7 nM	Serum samples	[108]
**Enoxacin (ENX)**	ENX-Dy^3+^-Ce^4+^-S_2_O_3_^2-^-H_2_SO_4_ system; (Additionally applied for: lomefloxacin, ofloxacin, norfloxacin, gatifloxacin)	CL excitation/482 nm, 578 nm	LOD = 624.3 nM	Biological fluids	[109]
	ENX- Dy^3+^- MnO_4_^-^- S_2_O_3_^2-^ -HNO_3_ system;	CL excitation / 482 nm, 578 nm	LOD = 686.8 nM	Biological fluids	[125]
	ENX-Tb^3+^-Na_2_SO_3_ system; pH = 7	ECL excitation /490 nm, 545 nm, 585 nm, 620 nm.	LOD = 0.054 nM	Dosage forms, urine samples.	[106]
	ENX-Tb^3+^-KMnO_4_-Na_2_SO_3_ (Additionally applied for ofloxacin)	CL excitation /490 nm, 545 nm, 585 nm, 620 nm.	LOD = 0.24 nM	Dosage forms, urine sample	[126]
	ENX-Tb^3+^- acetylacetone NPs pH = 7.2, phosphate buffer	345 nm/559 nm (FRET method)	LOD = 30 nM	Dosage forms, urine sample	[127]
**Enrofloxacin (EF)**	EF-Eu^3+^-phen system; pH = 9- acetate-ammonia buffer; micellar solution of DDBS.	330 nm/615 nm	LOD = 230 nM	Blood sample	[91]
	EF-Eu^3+^-optical sensor pH = 7.8	395 nm/617 nm	LOD = 75 nM	Pharma-ceutical tablets and serum samples	[128]
	EF-Eu^3+^-polymer nanofilament-based optical sensor pH = 7.5, HEPES buffer	340 nm/612 nm	LOD = 580 nM	Water-rich media	[129]
	EF-Tb^3+^-SLS system; pH = 6-acetic acid-sodium acetate buffer;	300 nm- 325 nm/549 nm	LOD = 5.56 nM	Chicken and trout muscle sample	[115]
	EF-Tb^3+^-Ag NPs pH = 6, Tris–HCl buffer	327 nm/545 nm	LOD = 58.4 nM LOQ = 191.9 nM	Milk samples	[130]
	EF-Tb^3+^-Fe(II)/(III)-H_2_O_2_ EF-Tb^3+^-KNO_2_-H_2_O_2_ (Additionally applied for levofloxacin and pefloxacin)	CL excitation/440 nm 515 nm	LOD = 120 nM LOD = 240 nM	Pharma-ceutical forms and urine samples	[131]
	EF-Tb^3+^-calf thymus DNA pH = 6.5-hexamethylenetetramine buffer	324 nm/ 546 nm	LOD = 6.95 nM	Beef serum	[132]
**Fleroxacin (FLX)**	FLX-Dy^3+^-MnO_4_^−^-S_2_O_3_^2−^-H_6_P_4_O_13_ system; (Additionally applied for pefloxacin and pipemidic acid)	CL excitation/482 nm, 578 nm	LOD = 0.812 nM	Foods, biological samples	[104]
	FLX-Dy^3+^-KMnO_4_-Na_2_S_2_O_3_-H_6_P_4_O_13_	CL excitation/482 nm, 578 nm	LOD = 0.3 nM	Injections and urine sample	[133]
	FLX-Eu^3+^-[(Me)_4_N]_2_[Pb_6_K_6_(m-BDC)_9_(OH)_2_]·H_2_O, where 1,3-H_2_BDC = 1,3-benzenedicarboxylic acid)	377 nm/612 nm	LOD = 118.8 nM	Human serum and urine	[134]
	FLX-Tb^3+^ photochemical fluorimetric system pH = 5.7, acetate buffer	320 nm/545 nm	LOD = 12 nM	Human urine samples	[135]
**Flumequine (FLU)**	FLU-Tb^3+^ Ce^4+^-Na_2_SO_3_-H_2_SO_4_ system;	Excitation by redox reaction/-	LOD = 382.7 nM; LOQ = 1148.1 nM;	Waste water samples	[105]
	FLU-Tb^3+^-SDS micelles system; pH = 7.6-Tris buffer;	340 nm/545 nm	LOD = 210.5 nM	Chicken muscle and liver, whole milk.	[136]
	FLU-Tb^3+^-1,10-phenanthroline in micellar SDS solutions pH = 7.5-acetate-ammonia buffer	330 nm/545 nm	LOD = 4.9 nM	Chicken meat	[137]
	FLU-Eu^3+^-Tb^3+^-nanocomposites pH = 6.9 acetic acid buffered with ammonia	255 nm/360 nm	LOD = 4.6 nM	Meat samples	[138]
**Garenoxacin (GAR)**	GAR-Tb^3+^-SDS micelles-Na_2_SO_3_ system; pH = 4.1-acetate buffer;	281 nm/546 nm	LOD = 46.9 nM; LOQ = 152.4 nM	Serum and urine samples	[89]
**Gatifloxacin (GFLX)**	GFLX- Eu^3+^ system in acetonitrile; pH = 3.5- acetate buffer;	395 nm/617 nm	LOD = 16 nM; LOQ = 28 nM	Pharma- ceutical tablets, serum.	[122]
	GFLX-Eu^3+^-SDBS system pH = 7.5-Tris–HCl buffer	338 nm/617 nm	LOD = 1 nM	Injections and human urine/serum samples	[139]
	GFLX-Eu^3+^ system in sol- gel matrix; pH = 6 - borate buffer;	370 nm/617 nm;	LOD = 0.16 nM	Pharma- ceutical and serum samples.	[140]
	GFLX-Tb^3+^ system in sol-gel matrix; pH = 3.5-acetate buffer;	350 nm/545 nm;	LOD = 20 nM	Pharma- ceutical and serum samples.	[140]
**Grepafloxacin (GREP)**	GREP-Tb^3+^-SDS micellar solution system; pH = 6-acetate buffer;	275 nm/546 nm	LOD = 27824.5 nM; LOQ = 83473.5 nM	Human serum and urine	[88]
	GREP-Tb^3+^-Ce(IV)–Na_2_SO_3_ pH = 4-15mM sodium dodecyl sulphate solution	280 nm/450 nm	LOD = 27.8 nM	Pharmaceutical tablets and human urine	[141]
**Levofloxacin (LEVO)**	LEVO-Tb^3+^ system; micellar solution of SDS; pH = 6-acetate buffer;	292 nm/546 nm	LOD = 27672.6 nM; LOQ = 83017.8 nM	Pharma- ceutical tablets, human serum, urine.	[87]
	LEVO-Tb^3+^ system; colloidal silver NPs; pH = 9-tris-HCl buffer;	284 nm/545 nm	LOD = 7.19x10^-9^ nM	Serum samples, urine	[95]
	LEVO-Tb^3+^-gold NPs system; pH = 6.8 NaH_2_PO_4_/Na_2_HPO_4_ buffer;	373 nm/545 nm	LOD = 0.21 nM; LOQ = 0.72 nM	Powder, tablets.	[99]
	LEVO-Sm^3+^ system; pH = 6-acetate buffer;	312 nm/553 nm	LOD = 52.5 nM; LOQ = 154.7 nM	Powder, tablets	[142]
	LEVO-Eu^3+^-Ce^4+^-SO_3_^2−^-H_2_SO_4_ system; (Additionally applied for moxifloxacin and trovafloxacin)	-	LOD = 276.7 nM; LOQ = 968.45 nM	Tablets	[110]
	LEVO-Eu^3+^-covalent organic framework-based hybrid material	380 nm/613 nm	LOD = 200 nM	Serum and urine samples	[143]
	LEVO-Ce(IV) in micellar solutions of of cetyltrimethyl ammonium bromide	250 nm/355 nm (FRET method)	-	Injections	[144]
**Lomefloxacin (LOM)**	LOM-Sm^3+^ system; pH = 6- acetate buffer;	310 nm/556 nm	LOD = 65.4 nM; LOQ = 199 nM	Powder, tablets.	[142]
	LOM-Tb^3+^-silver NPs system; pH = 6-acetate buffer;	274 nm/545 nm	LOD = 0.11 nM;	Tablets, serum, urine samples.	[98]
	LOM-Tb^3+^-Ce(IV)-Na_2_SO_3_ pH = 2-H_2_SO_4_	CL excitation/490 nm, 545 nm, 585 nm, 620 nm	LOD = 1.1 nM	Pharmaceutical tablets, urine and serum samples	[145]
**Marbofloxacin (MAR)**	MAR-Tb^3+^ system; pH = 6.0-acetic acid/ ammonium acetate buffer; (Additionally applied for: ciprofloxacin, danofloxacin, enrofloxacin, sarafloxacin, difloxacin, oxolinic acid, flumequine)	FL: 340 nm/545 nm; TR: 281 nm/545 nm	LOD = 165.56 nM (FL); LOD = 262.17 nM (TR);	Whole, semi-skimmed, skimmed milk.	[146]
	MAR-Tb_4_O_7_ NPs-TOPO-SDS-hexamine system; pH = 7.0–7.5-Tris buffer; (Applied also for: ciprofloxacin, danofloxacin, enrofloxacin, sarafloxacin, oxolinic acid, fumequine).	340 nm/545 nm.	LOD = 96.59 nM	Skimmed, semi- skimmed, whole milk samples.	[97]
**Moxifloxacin (MOXI)**	MOXI-Tb^3+^ system; colloidal silver NPs; pH = 9-Tris-HCl buffer;	284 nm/545 nm	LOD = 8.47 × 10^−9^ nM	Serum samples, urine	[95]
**Norfloxacin (NOR)**	NOR-Tb^3+^ -TOPO system pH = 5.5- acetate buffer; micellar solution of CPCl	333 nm/490 nm	LOD = 1.7 nM	Serum samples	[85]
**Marbofloxacin (MAR)**	MAR-Tb^3+^ system; pH = 6.0-acetic acid/ ammonium acetate buffer; (Additionally applied for: ciprofloxacin, danofloxacin, enrofloxacin, sarafloxacin, difloxacin, oxolinic acid, flumequine)	FL: 340 nm/545 nm; TR: 281 nm/ 545 nm	LOD = 165.56 nM (FL); LOD = 262.17 nM (TR);	Whole, semi-skimmed, skimmed milk.	[146]
	MAR-Tb_4_O_7_ NPs-TOPO-SDS-hexamine system; pH = 7.0–7.5-Tris buffer; (Applied also for: ciprofloxacin, danofloxacin, enrofloxacin, sarafloxacin, oxolinic acid, fumequine).	340 nm/545 nm.	LOD = 96.59 nM	Skimmed, semi-skimmed, whole milk samples.	[97]
**Moxifloxacin (MOXI)**	MOXI-Tb^3+^ system; colloidal silver NPs; pH = 9-Tris-HCl buffer;	284 nm/545 nm	LOD = 8.47 × 10^−9^ nM	Serum samples, urine	[95]
**Norfloxacin (NOR)**	NOR-Tb^3+^-TOPO system pH = 5.5-acetate buffer; micellar solution of CPCl	333 nm/490 nm	LOD = 1.7 nM	Serum samples	[85]
	NOR-Tb^3+^-calf thymus DNA; pH = 5.5-acetic acid–sodium acetate buffer	272 nm/545 nm	LOD = 35.8 nM	Eye-ear pharma-ceutical dosage forms	[121]
	NOR-Tb^3+^-zeolite of the CaA-type pH = 7-buffered with a 40% aqueous solution of urothropine (Additionally applied for ciprofloxacin)	365 nm/545 nm	LOD = 3131 nM	Urine and human plasma samples	[147]
	NOR-Tb^3+^-Na_2_SO_3_ system pH = 7	ECL excitation/490 nm, 545 nm, 585 nm, 620 nm	LOD = 0.028 nM	Pharmaceutical capsules, urine samples	[148]
	NOR-Sm^3+^ system; pH = 6-acetate buffer;	314 nm/553 nm	LOD = 84.5 nM; LOQ = 253.6 nM;	Powder, tablets	[142]
	NOR-Tb^3+^ sodium tetradecylsulfate system; pH = 7–8;	337 nm/545 nm	LOD = 0.0031 nM	Used in HELC	[149]
	NOR-Ce^4+^ system; Acidic pH-perchloric acid 0.02-0.08 mol/L;	-/550 nm	LOD = 31.3 nM; LOQ = 93.9 nM	Pharma-ceutical capsules, eye drops, urine	[100]
	NOR-Tb^3+^(bipy)_2_ (DPA)-K_2_S_2_O_8_ system; pH = 10-NaBO_4_-NaOH buffer;	ECL excitation /485 nm, 545 nm, 582 nm, 621 nm	LOD = 0.69 nM	Urine samples	[101]
	NOR- PEG coated Tb^3+^ doped ZnS NPs system; pH = 7.6-tris-HCl buffer;	334 nm/491 nm, 545 nm	LOD = 0.05 nM; LOQ = 0.17 nM	Eye drops, urine samples	[96]
	NOR-Tb^3+^ system flow through solid phase system; pH = 5.6-acetate buffer;	273 nm/545 nm.	LOD = 4.7 nM; LOQ = 15.7 nM	Serum and urine samples.	[150]
	NOR-Tb^3+^-KMnO_4_ -Na_2_SO_3_ system-optical flow-through sensor; pH = 3-KH phthalate-HCl buffer;	CL excitation/545 nm	LOD = 8.7 nM	Pharma-ceutical samples	[102]
	NOR-AMP-Tb^3+^ CPNSs system; pH = 7.5; (Additionally applied for: pefloxacin, sparfloxacin, fleroxacin, nalidixic acid as well.)	280 nm/545 nm	LOD = 10 nM	Milk	[93]
	NOR-Tb^3+^-SDS-Na_2_SO_3_ system; pH = 6.5-acetate buffer;	271 nm/545 nm;	LOD = 1.88 nM	Serum samples.	[108]
	NOR-Tb^3+^ system in sol-gel matrix; pH = 3.5-acetate buffer;	395 nm/545 nm;	LOD = 10 nM	Pharma-ceutical and serum samples.	[140]
	NOR- Eu^3+^ system in sol-gel matrix; pH = 6- borate buffer;	340 nm/617 nm;	LOD = 3.0 nM	Pharma-ceutical and serum samples.	[140]
	NOR fluorescence immunoassay coating-antigen-modified polystyrene particles - anti-norfloxacin monoclonal antibody conjugated with carboxyl-functionalized NaYF_4_:Yb,Er upconversion NPs pH = 7.4-phosphate-buffered saline	980 nm/542 nm	LOD = 0.03 nM	Milk, chicken, pork kidney samples	[151]
**Ofloxacin (OFLO)**	OFLO-Tb^3+^-TTDC; pH = 7–8;	337 nm/545 nm	LOD = 0.0000276 nM	Used in HELC	[149]
	Ce^4+^-Na_2_SO_3_-OFLO-Tb^3+^ system; Acidic pH- HCl. Flow-injection coupled with CL detection	-	LOD = 20.75 nM	Plasma samples	[152]
	OFLO-Ru(bipy)_2_(CIP)^2+^ Ce^4+^ system; Acidic pH -HNO_3_	CL excitation / 617 nm, 624 nm	LOD = 4.2 nM	Pharma-ceutical samples, urine samples	[103]
	OFLO-Eu^3+^ system; pH = 5.1- acetate buffer.	365 nm/617 nm	LOD = 3 nM; LOQ = 9 nM	Pharma-ceutical and serum samples	[153]
	OFLO-Tb^3+^-Na_2_SO_3_ system; pH = 7;	ECL excitation /490 nm, 545 nm, 585 nm, 620 nm.	LOD = 0.16 nM	Dosage forms, urine samples.	[106]
**Orbifloxacin (ORBI)**	ORBI-Tb^3+^ system; pH = 6-acetate buffer;	275 nm/545 nm	LOD = 8.35 nM; LOQ = 25.3 nM	Tablets, urine samples	[154]
**Pazufloxacin (PAZ)**	PAZ- Tb^3+^ system; pH = 6.3- acetate buffer;	330 nm/545 nm	LOD = 6.2 nM	Serum and urine samples	[155]
	PAZ-Eu^3+^-KMnO_4_- Na_2_S_2_O_4_ system; pH = 6.	CL excitation/592 nm, 617 nm, 695 nm.	LOD = 2.6 nM	Serum and urine samples.	[156]
	PAZ- Ce^4+^- Na_2_SO_3_- H_2_SO_4_ system;	-/ 247 nm	LOD = 2.2 nM	Urine samples	[157]
**Pefloxacin (PEF)**	PEF-Tb^3+^-TOPO system; pH = 5.5-acetate buffer; micellar solution of CPCl	333 nm/590 nm	LOD = 4.4 nM	Serum samples	[85]
	PEF-Tb^3+^- KMnO_4_/ H_2_SO_3_ system; Acidic pH-H_2_SO_4_;	CL excitation /543 nm	LOD = 41.1 nM	Semi-skimmed milk samples	[107]
	PEF-Tb^3+^-Ag NPs pH = 6-acetate buffer	273 nm/545 nm	LOD = 25 nM	Pharmaceutical capsules and serum samples	[158]
**Pipemidic acid (PPA)**	PPA-Tb^3+^-Ag^+^ NPs system; pH = 6-acetate buffer;	320 nm/545 nm.	LOD = 0.047 nM	Tablets, serum, urine samples.	[98]
	PPA-Tb^3+^ system; pH = 5.6- acetate buffer;	320 nm/545 nm.	LOD = 5.9 nM	Urine and serum samples	[159]
**Prulifloxacin (PUFX)**	PUFX-Tb^3+^-KMnO_4_-Na_2_S_2_O_4_ system; pH = 5.8; (Additionally applied for ulifloxacin –pH = 5.4)	CL excitation/490 nm, 545 nm, 585 nm, 620 nm.	LOD = 7 nM	Tablets	[160]
	PUFX-Tb^3+^-KMnO_4_-Na_2_SO_3_ KH_2_PO_4_-NaOH buffer	275 nm/423 nm	LOD = 8 nM	Pharmaceutical tablets, serum, and urine samples	[161]
**Ulifloxacin (UFX)**	UFX-Eu^3+^-SDBS system; pH = 8.6-NH_4_Cl/NH_3_x H_2_O buffer;	276 nm/616 nm.	LOD = 0.2 nM	Human serum and urine	[162]

LOD = limit of detection; LOQ = limit of quantification; Ad = adenine; AMP = adenosine monophosphate; Bipy = 2,2′-bipyridyl; CIP = 4-carboxyl-imidazole [4,5-*f*] [1,10]-phenanthroline; DPA = 2,6-pyridinedicarbocylic acid; CPCl = cetylpiridinium chloride; CL = chemiluminescence; ECL = electrochemiluminescence; NPs = nanoparticles; CPNPs = coordination polymer nanoparticles; CPNSs = coordination polymer nanosheets; CTAB = cetyltrimethylammonium bromide; DDBS = sodium dodecyl-benzensulfonate; DO3A = 1,4,7,10-tetraazacyclododecane-1,4,7-triacetic acid; FL = fluorescence; TR = time-resolved fluorescence; GMP = guanosine-5-monophosphate; HELC = high-efficiency liquid chromatography; HEPES = N-2-hydroxyethyl piperazine-*N*′-2-ethanesulfonic acid; Phen = 1,10-phenanthroline; SDS = sodium dodecyl sulphate; SDBS = sodium dodecylbenzene sulfonate; SLS = sodium laurylsulphate; TOPO = tri-n-octylphosphine oxide; TTDC = sodium tetradecylsulfate; FRET = fluorescence resonance energy transfer.

**Table 5:** Determination of miscellaneous ions using lanthanide–quinolone systems.

Ion	Quinolone	Conditions	λ_absorption_	LOD/LOQ	Ref
Er^3+^	Norfloxacin	-Cetylpyridinium chloride; -pH = 9.35-NH_4_Cl/NH_3_ buffer;	515 (+) nm, 517 (−) nm (second derivative spectrum)	LOD = 6.6 × 10^−6^ mol/L	[164]
Eu^3+^	Gatifloxacin	-SDBS micelle solution, La^3+^; -pH = 7-NH_4_Ac-NH_3_*H_2_O buffer;	λ_ex_ = 336 nm/ λ_em_ = 616 nm	LOD = 7 × 10^−14^ mol/L	[165]
Ho^3+^	Norfloxacin	-Cetylpyridinium chloride; -pH = 9.35-NH_4_Cl/ NH_3_ buffer;	444 (+) nm, 446 (−) nm (second derivative spectrum)	LOD = 6.6 × 10^-6^ mol/L	[164]
Nd^3+^	Norfloxacin	-Cetylpyridinium chloride; -pH = 9.35-NH_4_Cl/ NH_3_ buffer;	570 (+) nm, 568 (−) nm (second derivative spectrum)	LOD = 6.7 × 10^−6^ mol/L	[164]
Cu^2+^	1-methyl-4-hydroxy- 3-(N-2-ethyl-5- aminothiadazolyl-)- carbamoyl- quinoline-2-one	-pH = 7-MOPS buffer;	λ_ex_ = 320 nm/ λ_em_ = 547 nm	LOD = 4.3 × 10^−9^ mol/L	[166]
Sc^3+^	Nalidixic acid	-Method used for the extraction of Sc^3+^; -pH = 3.4; -dicholoromethane;		SF = 1.4 × 10^4^ (from Eu^3+^); SF = 3.6 × 10^3^ (from Nd^3+^)	[167]
H_2_PO_4_^−^ HPO_4_^2−^	4-Hydroxy-1-methyl-2-oxo-1,2-dihydro-quinoline-3-carboxylic acid-ethyl-[1,3,4]thiadiazol-2-yl) amide	-pH = 7.4-HEPES buffer; -Tb^3+^ complex-Triton X-100 system;	λ_ex_ = 320 nm/ λ_em_ = 548 nm	LOD = 110 × 10^−9^ mol/L	[168]

LOD = limit of detection; SF = separation factor.

**Table 6:** Determination of DNA using quinolone–lanthanide systems.

Type of Nucleic Acid	Ligand (L)	Lanthanide Ion (Ln^3+)^	Conditions (pH, L:Ln^3+^)	λex/λem	LOD/LOQ	Ref
fs-DNA	Quinolone derivative	Tb^3+^	-pH = 9-Tris- HCl buffer; -1:1;	340 nm/545 nm	12 ng/mL;	[172]
ct-DNA	Quinolone derivative	Tb^3+^	-pH = 9-Tris-HCl buffer; -1:1;	340 nm/545 nm	10 ng/mL	[172]
ct-DNA	Danoflaxacin	Tb^3+^	-pH = 7.8-Tris- HCl buffer;	347 nm/545 nm	8 ng/mL	[180]
hs-DNA	Prulifloxacin	Tb^3+^	-pH = 6.3-Tris-HCl buffer; -2:1;	345 nm/545 nm	2.1 ng/mL	[171]
cf-DNA	Gatifloxacin	Eu^3+^	-pH = 6.5-HMA-HCl buffer; -3.1:1;	331 nm/617 nm	6 × 10^−9^ g/mL	[174]
ds-DNA	Difloxacin	Tb^3+^	-pH = 7.4-MOPS buffer; -1:1;	340 nm/545 nm	0.5 ng/mL	[175]
ss-DNA	Difloxacin	Tb^3+^	-pH = 7.4-MOPS buffer; -1:1;	340 nm/545 nm	2 ng/mL	[175]
Oligo-nucleotides	Cs124-TPA- Tb NPs	Tb^3+^	-pH = 7.5;	328 nm/546 nm	8 × 10^−11^ mol/L	[179]
Oligo- nucleotides	DTPA-cs214-CF_3_-NCS; DTPA-cs124-NCS	Tb^3+^, Eu^3+^, Dy^3+^, Sm^3+^			0.5–1 × 10^−12^ mol/L	[177]

fs-DNA = fish sperm DNA; ct-DNA = calf thymus DNA; hs-DNA = hering sperm DNA; ds-DNA = double-stranded DNA; ss-DNA = single-stranded DNA; HMA = hexamethylenamine; NPs = nanoparticles.
